# Elevated visceral adiposity index linked to improved cognitive function in middle-aged and elderly Chinese: evidence from the China health and retirement longitudinal study

**DOI:** 10.3389/fnagi.2023.1270239

**Published:** 2023-10-20

**Authors:** Zhaohao Zeng, Kunyu Huang, Yanmei Cen, Wen Jin, Yingao Shen, Lijiao Xiong, Fengju Mao, Guo Hong, Yu Luo, Xiaoguang Luo

**Affiliations:** ^1^Department of Neurology, Shenzhen People’s Hospital (The Second Clinical Medical College, Jinan University; The First Affiliated Hospital, Southern University of Science and Technology), Shenzhen, Guangdong, China; ^2^The First Clinical Medical College of Jinan University, Guangzhou, Guangdong, China; ^3^Guangdong Provincial Clinical Research Center for Geriatrics, Shenzhen Clinical Research Center for Geriatrics, Shenzhen People's Hospital (The Second Clinical Medical College, Jinan University; The First Affiliated Hospital, Southern University of Science and Technology), Shenzhen, China; ^4^Department of Pharmacy, Shenshan Medical Center, Memorial Hospital of Sun Yat-sen University, Shanwei, Guangdong, China; ^5^Department of Geriatrics, Shenzhen People’s Hospital (The Second Clinical Medical College, Jinan University; The First Affiliated Hospital, Southern University of Science and Technology), Shenzhen, Guangdong, China

**Keywords:** cognitive function, visceral adiposity index, visceral obesity, China health and retirement longitudinal study, dementia

## Abstract

**Object:**

Cognitive decline and obesity are major global public health issues, and their association has been widely acknowledged. The link between the visceral adiposity index (VAI) and cognitive function in the Chinese population remains uncertain. This study aims to investigate the effects of VAI levels on cognitive function in the Chinese middle-aged and elderly population.

**Methods:**

We analyzed longitudinal data from the China Health and Retirement Longitudinal Study (CHARLS) collected in 2011, 2013, 2015, and 2018. VAI levels were divided into three tertiles. Generalized estimating equation (GEE) models were used to explore the relationships between VAI levels and cognitive function, including overall cognitive scores, episodic memory, and mental status. Adjustments were made for potential confounders.

**Results:**

The study consisted of 2,677 participants. Contrary to expectations, higher VAI levels were associated with higher overall cognitive scores and improved episodic memory scores, while no significant effect was observed on mental status. The GEE models consistently indicated that higher VAI levels were associated with higher overall cognitive scores, primarily due to their association with episodic memory. Stratified analyses revealed that the VAI was associated with better cognitive function primarily in males, individuals under 60 years old, those with lower education levels, rural residents, and married individuals, mainly in relation to episodic memory. No significant interactions were observed between VAI and demographic factors.

**Conclusion:**

Our findings suggest that higher visceral adiposity is associated with slower cognitive decline in the Chinese middle-aged and elderly population, especially in its association with episodic memory. These results underline the need to further investigate the potential protective role of visceral fat in cognitive function, potentially offering new insights for interventions to enhance cognitive function and prevent dementia in this population.

## 1. Introduction

Cognitive decline, as defined by the fifth edition of the Diagnostic and Statistical Manual of Mental Disorders (DSM-5), refers to the subjective and objective deterioration in one or more cognitive dimensions, including perception, attention, memory, thinking, language, judgment, and reasoning. It manifests when it affects daily instrumental activities or occurs alongside mental or other psychological conditions, ultimately leading to the development of dementia (Posar and Visconti, 2023). Cognitive decline has emerged as a pressing global public health issue. A study shows that around 55 million people are currently suffering from cognitive decline or dementia and this number may even increase to 139 million by 2050 according to proper estimation globally (Chowdhary et al., 2021). China, one of the countries with a large proportion of the world’s population, is also facing an increase in population in cognitive decline or dementia. A comprehensive cross-sectional study conducted in China found that among adults aged 60 years and older, the prevalence of cognition decline and dementia was 15.5 and 6.0%, respectively. In other words, approximately 15.07 million and 38.77 million individuals suffer from cognition decline and dementia, respectively (Jia et al., 2020). As we know, cognitive decline or impairment can indeed impose a significant burden on individuals, their families, and society as a whole, including healthcare costs, caregiver strain, economic impact, and social and emotional well-being. Hence we should raise attention to identify the risk factors of cognitive decline (Ren et al., 2022). Identifying risk factors for cognitive decline plays a crucial role in implementing strategies to prevent and slow down the progression of the disease. Several factors are recognized as potential risks for cognitive decline, such as age, level of education, hyperlipidemia, marital status, smoking, and the presence of certain chronic diseases such as diabetes, heart disease, and cerebrovascular disease (Jia et al., 2020). It is worth noting that many risk factors associated with obesity, such as hyperlipidemia, diabetes, heart disease, and cerebrovascular disease, are also closely related to cognitive decline.

Obesity refers to the state of being excessively overweight or obese, which is typically caused by an excessive accumulation of body fat, including total body obesity and central obesity (Anand et al., 2022; Panuganti et al., 2023). Body mass index (BMI) is a convenient and economical means of estimating obesity (BMI over 25 kg/m^2) that is in total obesity but fails to estimate central obesity efficiently (Anand et al., 2022). Obesity has emerged as a grave global health concern, this trend can be attributed to rapid economic development and shifts in lifestyle patterns (Ng et al., 2014; Afshin et al., 2017). Notably, China has witnessed a gradual increase in obesity prevalence, establishing itself as the country with the highest number of overweight and obese individuals worldwide (Wang Y. et al., 2021). In 2018, data from the Chinese Center for Disease Control and Prevention revealed that more than 8.1% of Chinese adults, equating to approximately 85 million individuals, were classified as obese (Wang L. et al., 2021). Furthermore, studies have indicated that obesity also shows a significant association with cognitive function (Dye et al., 2017). Previous studies have shown that obesity is associated with an increased risk of cognitive decline and dementia. However, some studies on the “obesity paradox” have found that using BMI or waist circumference as indicators of obesity, obesity seems to provide a certain level of protection for cognition (Qizilbash et al., 2015; Tang et al., 2021). In fact, it is unreasonable to assess obesity using BMI fails to indicate the distribution of obesity (Stevens et al., 2008). While waist circumference plays a role in identifying central obesity, it is limited in its ability to effectively assess the overall distribution of fat. These measures may not fully capture the health risks associated with abdominal obesity. The visceral adiposity index (VAI) is a scientifically devised mathematical model used to approximate the function and quantity of visceral fat within an individual’s body (Amato et al., 2010). Visceral fat, when present in increased amounts, has been linked to various health conditions such as metabolic syndrome, diabetes, cardiovascular diseases, and certain types of cancer (Kahn, 2005; Amato and Giordano, 2014). The VAI assessment method incorporates both anthropometric data, such as waist circumference, and metabolic markers, such as triglycerides and high-density lipoprotein cholesterol levels. In essence, the VAI offers a holistic assessment of a person’s visceral fat status. Previous studies demonstrate that higher visceral adipose is associated with poor cognitive functioning but relations are attenuated by age (Isaac et al., 2011; Yoon et al., 2012; Anand et al., 2022).

In our research, we intend to scrutinize the correlation between the VAI and cognitive function in Chinese individuals who are 45 years of age or older. The data for this study will be sourced from the China Health and Retirement Longitudinal Study (CHARLS). Employing longitudinal analyses, we will explore the connection between the VAI and cognitive performance. Moreover, we aim to understand how this association may be affected by varying factors such as age, gender, educational attainment, and the presence of certain chronic diseases. By shedding light on the intricate link between visceral fat and cognitive function, our study could potentially inform and enhance interventions designed to mitigate the risk of cognitive deterioration and dementia.

## 2. Methods

### 2.1. Study population and design

The data for this study were obtained from the CHARLS, an ongoing longitudinal survey that aims to capture the health conditions and related factors among community-dwelling adults aged 45 years and older across 28 provinces in China. CHARLS provides comprehensive information on personal characteristics, family dynamics, health status, cognitive function, retirement, and personal property status. It is a nationally representative survey that offers reliable data on middle-aged and older individuals.

The data used in our study were freely accessible to the general public and were collected through a series of exams conducted every 2 to 3 years. We utilized data from four waves: wave 1 (collected in 2011), wave 2 (collected in 2013), wave 3 (collected in 2015), and wave 4 (collected in 2018) of the CHARLS survey. These waves allowed for a longitudinal analysis of the study variables.

**Figure 1:**
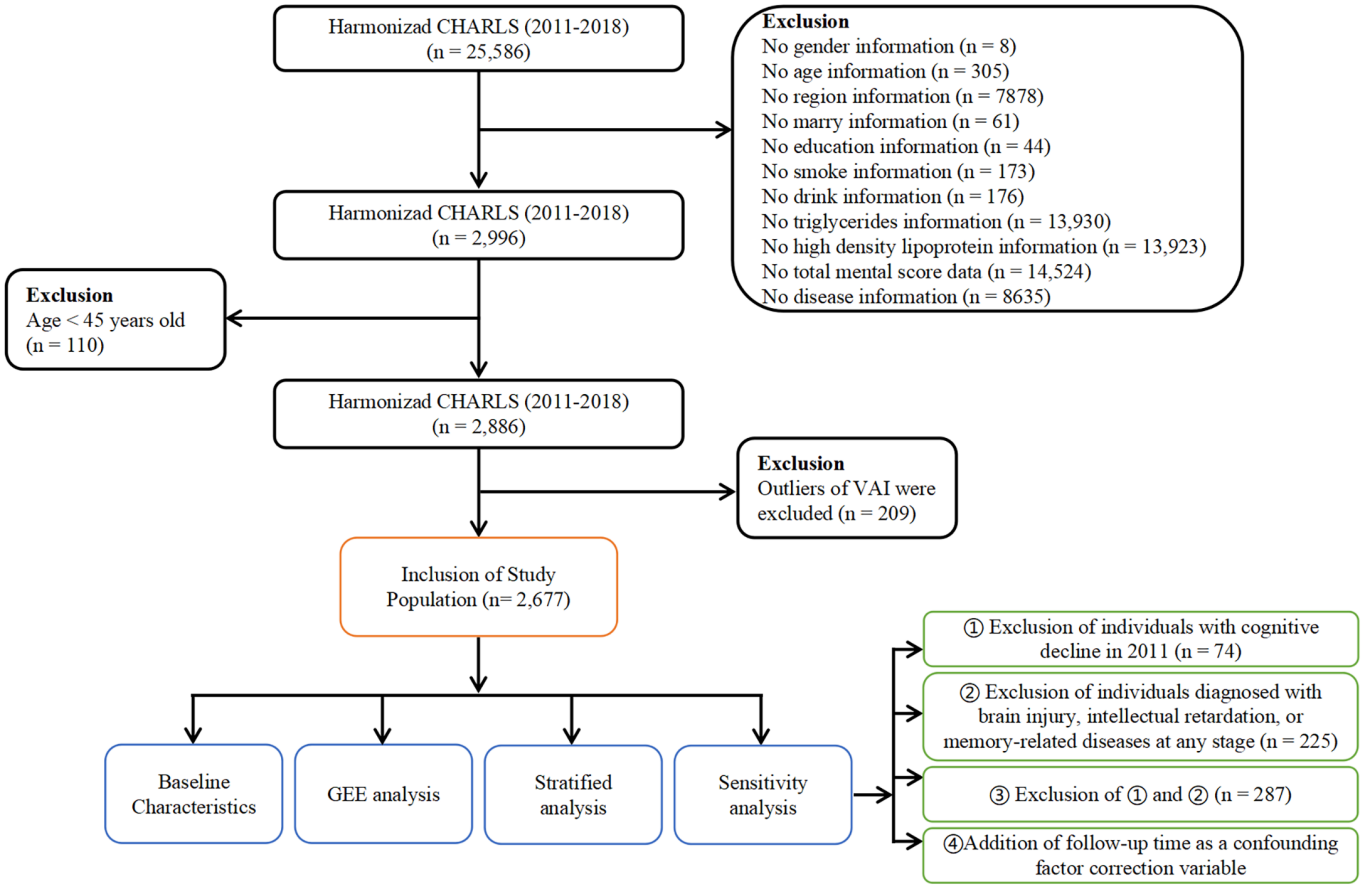
Flowchart of study population selection. Flowchart of the participants’ selection from CHARLS 2011 to 2018.

Out of the initial 25,586 participants enrolled, a total of 22,590 participants were excluded for reasons including missing essential information such as age, gender, region, etc. Additionally, 110 participants below the age of 45 were excluded. To ensure data quality, any participants with missing information or abnormal values for the VAI, as determined by the Tukey method based on VAI quartiles, were also excluded. After applying these exclusion criteria, a total of 2,677 eligible individuals remained in the final analysis sample. The selection process for participant inclusion is illustrated in Figure 1, providing a visual representation of the steps taken to arrive at the study sample used for analysis.

### 2.2. Calculation of VAI

VAI was calculated using gender-specific formulas proposed by Amato et al. (2010) to account for physiological differences between males and females in terms of visceral adiposity. VAI serves as an indicator of visceral adiposity and is derived from anthropometric and metabolic parameters. The formulas used for calculating the VAI were as follows: For males: VAI = (WC/(39.68 + 1.88 × BMI)) × (TG/1.03) × (1.31/HDL), For females: VAI = (WC/(36.58 + 1.89 × BMI)) × (TG/0.81) × (1.52/HDL). Here, WC represents waist circumference, BMI is body mass index, TG stands for triglycerides, and HDLC represents high-density lipoprotein cholesterol. These formulas allowed us to calculate gender-specific VAI values, providing a more accurate assessment of visceral adiposity for males and females. The calculated VAI values were subsequently divided into tertiles, yielding three levels: Tertile 1 (T1), Tertile 2 (T2), and Tertile 3 (T3). The corresponding ranges for these levels in our study were “< 2.36,” “2.36–4.54,” and “> 4.54.”

### 2.3. Cognitive score assessment

Cognitive function assessment in the CHARLS database included measures of global cognitive score, episodic memory, and mental status. Episodic memory assessment involved the presentation of a list of Chinese words to participants, who were then asked to immediately recall as many nouns as possible. After a 5-min delay, participants were prompted to recall the nouns again (delayed recall). Episodic memory scores were computed as the sum of the immediate and delayed recall scores, ranging from 0 to 20. A higher episodic memory score indicated better performance in remembering and recalling the presented nouns. Mental status was evaluated using the Telephone Interview of Cognitive Status (TICS) questionnaire. Participants were asked to perform several tasks, including subtracting 7 from 100 multiple times, naming the current date, and redrawing a previously shown picture. The correct responses were tallied to calculate a single TICS score ranging from 0 to 11. A higher TICS score reflected better mental status and cognitive functioning. The global cognitive score was determined by summing the episodic memory score and the TICS score. This composite score provided an overall measure of cognitive function, ranging from 0 to 31. Higher global cognitive scores indicated better overall cognitive performance, considering both episodic memory and mental status. Cognitive assessments, including episodic memory and mental status, were conducted by trained investigators following standardized protocols. These assessments, along with the calculation of the global cognitive score, enabled a comprehensive evaluation of cognitive function within the CHARLS study population.

### 2.4. Assessment of other variables

Data on various demographic and clinical characteristics were obtained for the participants from the CHARLS public database. These included age, gender, education, marital status, region (urban or rural), drinking and smoking status, as well as self-reported or physician-diagnosed chronic diseases such as cancer, hypertension, diabetes, heart disease, dyslipidemia, stroke, liver disease, kidney disease, and digestive disease. Education was categorized into three levels based on the official coding file of the CHARLS. Level 1 represented individuals with no formal education (illiterate), those who did not complete primary school but could read, those who received private tutoring (Sishu), or those who completed elementary or middle school. Level 2 included individuals who completed high school or vocational school. Level 3 encompassed respondents with two/three-year college education, a college degree, or a postgraduate degree.

### 2.5. Statistical analysis

Descriptive statistics were used to summarize the baseline characteristics of the study population. Continuous variables were assessed for normality using the Shapiro–Wilk test. For variables that were found to be non-normally distributed, we chose to report the median and interquartile range (IQR), whereas normally distributed variables were represented using the mean and standard deviation. Categorical variables were presented as counts and percentages. One-way analysis of variance (ANOVA) was used to compare the differences in continuous variables, while the Chi-square test was employed to compare the differences in categorical variables among the three groups. The relationship between the VAI and cognitive scores was assessed using generalized estimating equation (GEE) models. Four different models were employed to examine this association, with each model incorporating specific adjustments: Model 1: Adjusted for the cognitive score in 2011. Model 2: Further adjusted for gender and age. Model 3: In addition to the adjustments in Model 2, further adjustments were made for education, marital status, smoking, and drinking. Model 4: In addition to the adjustments in Model 3, further adjustments were made for hypertension, diabetes, heart disease, stroke, dyslipidemia, liver disease, kidney disease, and digestive system disease. Furthermore, four sensitivity analyses were performed to evaluate the robustness of the findings: 1. Exclusion of individuals with cognitive decline in 2011. 2. Exclusion of individuals with brain injury, intellectual retardation, or memory-related diseases, along with those having cognitive decline in 2011. 3. Combination of exclusion of individuals with cognitive decline in 2011 and individuals with brain injury, intellectual retardation, or memory-related diseases. 4. Addition of follow-up time as a confounding factor correction variable. Model 5 is reported for the fourth sensitivity analysis. In the sensitivity analysis, individuals with low cognitive function in 2011 were excluded from the study population. Low cognitive function was defined as having a cognitive total score below two standard deviations from the mean baseline cognitive score. Participants whose cognitive total score in 2011 fell below the threshold of two standard deviations below the mean baseline cognitive score were classified as having low cognitive function. All statistical analyses were conducted using R version 4.1.2. A significance level of *p* < 0.05 was considered statistically significant, indicating evidence of a meaningful association.

## 3. Results

### 3.1. Baseline characteristics of study participants stratified by VAI tertiles

In this research, we scrutinized data from 2,677 subjects, segregating them into three categories based on the tertiles of their VAI: T1, T2, and T3. As shown in Table 1, the average age in the T1 group was 57.3 years (Standard deviations (SD) = 8.10), in the T2 group it was 56.3 years (SD = 7.65), and in the T3 group it was 56.0 years (SD = 7.48). We observed a significant age difference among these groups (*p* = 0.001), with an evident declining pattern (*P* for trend = 0.001) as we moved from lower to higher VAI groups. When considering gender, the percentage of females escalated from T1 (28.6%) to T2 (46.9%) and T3 (57.5%), whereas the male percentage displayed a converse trend. The gender differences were statistically significant (*p* < 0.001), and a distinct trend was observed (*P* for trend<0.001). With respect to variables such as education, marital status, and geographic region, no significant discrepancies or patterns were discernible among the VAI groups. Nevertheless, lifestyle habits, specifically drinking and smoking, presented notable differences and patterns across the groups (*p* < 0.001 for both). The data indicated a declining ratio of drinkers and smokers in the higher VAI groups. Finally, as for health conditions including hypertension, diabetes, heart disease, and dyslipidemia, we found significant differences and patterns among the VAI groups (all *p* < 0.001), suggesting a greater incidence of these conditions in the higher VAI groups. Other health conditions like cancer, stroke, liver disease, kidney disease, and digestive disease did not exhibit significant differences or patterns across the groups, except for digestive disease which did show a significant pattern (*P* for trend = 0.04).

**Table 1:** Baseline characteristics of the study population.

	VAI				*p* value	*p* for trend
	Total	T1	T2	T3		
	*N* = 2,677	*N* = 892	*N* = 893	*N* = 892		
Age	56.5 (7.77)	57.3 (8.10)	56.3 (7.65)	56.0 (7.48)	0.001	0.001
Gender					<0.001	<0.001
Female	1,187 (44.3%)	255 (28.6%)	419 (46.9%)	513 (57.5%)		
Male	1,490 (55.7%)	637 (71.4%)	474 (53.1%)	379 (42.5%)		
Education					0.678	0.316
1. Less than lower secondary	2,238 (83.6%)	735 (82.4%)	751 (84.1%)	752 (84.3%)		
2. Upper secondary & vocational training	395 (14.8%)	143 (16.0%)	125 (14.0%)	127 (14.2%)		
3. Tertiary	44 (1.64%)	14 (1.57%)	17 (1.90%)	13 (1.46%)		
Marry					0.309	0.301
Married	2,493 (93.1%)	830 (93.0%)	826 (92.5%)	837 (93.8%)		
Widowed	151 (5.64%)	48 (5.38%)	57 (6.38%)	46 (5.16%)		
Separated	8 (0.30%)	5 (0.56%)	0 (0.00%)	3 (0.34%)		
Divorced	15 (0.56%)	5 (0.56%)	5 (0.56%)	5 (0.56%)		
Never married	10 (0.37%)	4 (0.45%)	5 (0.56%)	1 (0.11%)		
Drinking					<0.001	<0.001
No	966 (36.1%)	225 (25.2%)	346 (38.7%)	395 (44.3%)		
Yes	1711 (63.9%)	667 (74.8%)	547 (61.3%)	497 (55.7%)		
Smoke					<0.001	<0.001
No	1,351 (50.5%)	348 (39.0%)	473 (53.0%)	530 (59.4%)		
Yes	1,326 (49.5%)	544 (61.0%)	420 (47.0%)	362 (40.6%)		
Region					0.003	0.001
Rural village	1,579 (59.0%)	558 (62.6%)	533 (59.7%)	488 (54.7%)		
Urban community	1,098 (41.0%)	334 (37.4%)	360 (40.3%)	404 (45.3%)		
Cancer					0.442	0.219
No	2,661 (99.4%)	889 (99.7%)	887 (99.3%)	885 (99.2%)		
Yes	16 (0.60%)	3 (0.34%)	6 (0.67%)	7 (0.78%)		
Hypertension					<0.001	<0.001
No	2005 (74.9%)	724 (81.2%)	682 (76.4%)	599 (67.2%)		
Yes	672 (25.1%)	168 (18.8%)	211 (23.6%)	293 (32.8%)		
Diabetes					<0.001	<0.001
No	2,521 (94.2%)	858 (96.2%)	848 (95.0%)	815 (91.4%)		
Yes	156 (5.83%)	34 (3.81%)	45 (5.04%)	77 (8.63%)		
Heart disease					<0.001	<0.001
No	2,334 (87.2%)	809 (90.7%)	775 (86.8%)	750 (84.1%)		
Yes	343 (12.8%)	83 (9.30%)	118 (13.2%)	142 (15.9%)		
Stroke					0.858	0.855
No	2,631 (98.3%)	877 (98.3%)	876 (98.1%)	878 (98.4%)		
Yes	46 (1.72%)	15 (1.68%)	17 (1.90%)	14 (1.57%)		
Dyslipidemia					<0.001	<0.001
No	2,392 (89.4%)	832 (93.3%)	819 (91.7%)	741 (83.1%)		
Yes	285 (10.6%)	60 (6.73%)	74 (8.29%)	151 (16.9%)		
Liver disease					0.901	0.694
No	2,587 (96.6%)	863 (96.7%)	864 (96.8%)	860 (96.4%)		
Yes	90 (3.36%)	29 (3.25%)	29 (3.25%)	32 (3.59%)		
Kidney Disease					0.585	0.314
No	2,520 (94.1%)	834 (93.5%)	842 (94.3%)	844 (94.6%)		
Yes	157 (5.86%)	58 (6.50%)	51 (5.71%)	48 (5.38%)		
Digestive disease					0.052	0.040
No	2084 (77.8%)	683 (76.6%)	682 (76.4%)	719 (80.6%)		
Yes	593 (22.2%)	209 (23.4%)	211 (23.6%)	173 (19.4%)		

Characteristics of study participants categorized by VAI tertiles at baseline. Continuous variables are presented as mean (median) and SD (IQR), while categorical variables are represented as N (percentage). ANOVA was used to compare the differences in continuous variables, while the Chi-square test was employed to compare the differences in categorical variables among the three groups.

### 3.2. Baseline cognitive scores across four waves stratified by VAI tertiles

**Figure 2:**
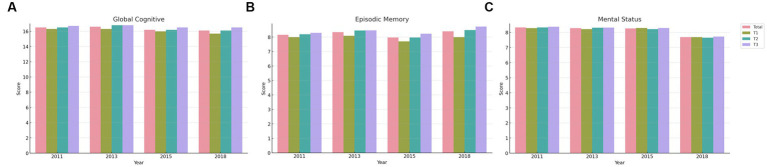
Longitudinal changes in cognitive scores over four waves. Wave 1 (collected in 2011), wave 2 (collected in 2013), wave 3 (collected in 2015), and wave 4 (collected in 2018) of the CHARLS survey.

Our observations across each wave indicated a slight increase in cognitive scores as we transitioned from the lower VAI group (T1) to the higher VAI groups (T2, T3). As shown in Table 2, in the first wave, for instance, the global cognitive score saw a slight increase from T1 (mean = 16.3, SD = 4.04) to T3 (mean = 16.7, SD = 3.94). This trend was consistent in the second wave as well, with scores marginally rising from T1 (mean = 16.3, SD = 3.95) to T3 (mean = 16.8, SD = 3.87). Similar patterns emerged across all waves and all three cognitive measures (As shown in Figure 2). Overall, these results suggest a potential association between VAI and cognitive function, particularly in terms of global cognition and episodic memory.

**Table 2:** Baseline cognitive scores of the study population: comparison across four waves.

	VAI				*p* value	*p* for trend
	Total	T1	T2	T3		
*Global cognitive*						
Wave 1	16.5 (4.00)	16.3 (4.04)	16.5 (4.02)	16.7 (3.94)	0.105	0.036
Wave 2	16.6 (3.93)	16.3 (3.95)	16.8 (3.96)	16.8 (3.87)	0.014	0.009
Wave 3	16.2 (3.99)	16.0 (4.08)	16.2 (3.99)	16.5 (3.90)	0.015	0.004
Wave 4	16.1 (4.90)	15.7 (4.94)	16.1 (4.85)	16.5 (4.88)	0.003	0.001
*Episodic memory*						
Wave 1	8.16 (3.17)	7.99 (3.18)	8.20 (3.24)	8.29 (3.08)	0.122	0.046
Wave 2	8.34 (3.12)	8.09 (3.10)	8.46 (3.16)	8.47 (3.08)	0.012	0.009
Wave 3	7.97 (3.19)	7.70 (3.31)	7.97 (3.13)	8.24 (3.10)	0.002	<0.001
Wave 4	8.40 (3.90)	7.99 (3.92)	8.49 (3.88)	8.73 (3.86)	<0.001	<0.001
*Mental status*						
Wave 1	8.33 (1.75)	8.28 (1.74)	8.33 (1.74)	8.38 (1.77)	0.500	0.239
Wave 2	8.28 (1.77)	8.22 (1.80)	8.31 (1.76)	8.32 (1.76)	0.456	0.251
Wave 3	8.27 (1.71)	8.29 (1.66)	8.22 (1.75)	8.29 (1.71)	0.632	0.967
Wave 4	7.69 (1.97)	7.69 (1.96)	7.65 (1.98)	7.73 (1.97)	0.725	0.700

Characteristics of study participants categorized by VAI tertiles at baseline. Continuous variables are presented as mean (median) and SD (IQR), while categorical variables are represented as N (percentage). ANOVA was used to compare the differences in continuous variables, while the Chi-square test was employed to compare the differences in categorical variables among the three groups.

### 3.3. Gee analysis of the association between VAI tertiles and cognitive scores

In our study, we employed generalized estimating equations to investigate the influence of the VAI on three cognitive scores: global Cognitive, episodic memory, and mental status. As shown in Table 3, For global cognitive scores, all models showed a significant positive association with VAI. In Model 1, which adjusted for the cognitive score in 2011, the regression coefficient (β) for VAI was 0.074 (95% CI: 0.034 to 0.113, *p* < 0.001). This association remained significant after further adjusting for gender and age in Model 2 (β = 0.067, 95% CI: 0.027 to 0.106, *p* < 0.001), and additional adjustments for education, marital status, smoking, and drinking in Model 3 (β = 0.070, 95% CI: 0.031 to 0.108, *p* < 0.001). The significance persisted in Model 4, which further adjusted for hypertension, diabetes, heart disease, stroke, dyslipidemia, liver disease, kidney disease and digestive diseases (β = 0.064, 95% CI: 0.025 to 0.104, *p* = 0.001). For episodic memory scores, the association with VAI was also significant across all models, with the highest β in Model 1 (β = 0.071, 95% CI: 0.039 to 0.104, *p* < 0.001) and the lowest β in Model 4 (β = 0.051, 95% CI: 0.019 to 0.082, *p* = 0.002). However, for mental status scores, the association with VAI was not significant across all models.

**Table 3:** Association between VAI and cognitive function: GEE analysis.

	VAI								*p* for trend
	Total		TI		T2		T3		
	β (95% CI)	*p* value	β (95% CI)	*p* value	β (95% CI)	*p* value	β (95% CI)	*p* value	
*Global cognitive*									
Model 1	0.074 (0.034,0.113)	<0.001	0	Ref	0.248 (−0.024, 0.519)	0.074	0.408 (0.135, 0.682)	0.004	0.003
Model 2	0.067 (0.027, 0.106)	<0.001	0	Ref	0.200 (−0.071, 0.464)	0.149	0.353 (0.078, 0.628)	0.012	0.012
Model 3	0.070 (0.031, 0.108)	<0.001	0	Ref	0.224 (0.038, 0.486)	0.094	0.370 (0.101, 0.639)	0.007	0.007
Model 4	0.064 (0.025, 0.104)	0.001	0	Ref	0.214 (−0.048, 0.476)	0.109	0.330 (0.057, 0.603)	0.018	0.018
*Episodic memory*									
Model 1	0.071 (0.039, 0.104)	<0.001	0	Ref	0.300 (0.077,0.516)	0.008	0.438 (0.218,0.658)	<0.001	<0.001
Model 2	0.052 (0.021, 0.084)	0.001	0	Ref	0.197 (−0.015,0.409)	0.069	0.304 (0.087,0.521)	0.006	0.006
Model 3	0.054 (0.023, 0.085)	<0.001	0	Ref	0.216 (0.008, 0.425)	0.042	0.312 (0.098,0.525)	0.004	0.004
Model 4	0.051 (0.019, 0.082)	0.002	0	Ref	0.213 (0.005, 0.421)	0.045	0.289 (0.074,0.504)	0.009	0.008
*Mental status*									
Model 1	0.005 (−0.011, 0.022)	0.520	0	Ref	−0.027 (−0.141,0.087)	0.640	−0.002 (−0.112, 0.116)	0.970	0.968
Model 2	0.017 (−0.001, 0.034)	0.041	0	Ref	0.021 (−0.094,0.135)	0.725	0.080 (−0.036, 0.197)	0.175	0.174
Model 3	0.018 (0.002, 0.034)	0.031	0	Ref	0.025 (−0.088, 0.138)	0.665	0.084 (−0.030, 0.199)	0.147	0.146
Model 4	0.014 (−0.002, 0.031)	0.092	0	Ref	0.018 (−0.096,0.130)	0.759	0.059 (−0.058, 0.175)	0.325	0.325

Model 1: Adjusted for the cognitive score in 2011. Model 2: Further adjusted for gender and age. Model 3: In addition to the adjustments in Model 2, further adjustments were made for education, marital status, smoking, and drinking. Model 4: In addition to the adjustments in Model 3, further adjustments were made for hypertension, diabetes, heart disease, stroke, dyslipidemia, liver disease, kidney disease, and digestive system disease.

### 3.4. Stratified analysis of the association between VAI tertiles and cognitive scores

In the stratified analysis, we further investigated the effect of the VAI on global cognitive (Table 4), episodic memory (Table 5), and mental status scores (Table 6), using T1 as the reference group. The analysis was adjusted for cognitive scores from 2011 and potential confounders such as hypertension, diabetes, tumors, heart diseases, stroke, and dyslipidemia. For global cognitive scores, the gender-stratified analysis showed a significant positive association with VAI for male participants in both the T2 (β = 0.424, *p* = 0.021) and T3 (β = 0.598, *p* = 0.003) groups. No significant association was found for female participants in either group (T2: β = −0.056, *p* = 0.836; T3: β = 0.053, *p* = 0.842). The age-stratified analysis showed a significant positive association in the T3 group for participants aged <60 years (β = 0.406, *p* = 0.030). In the education-stratified analysis, both education groups in the T3 group showed significant associations (Education 1: β = 0.567, *p* < 0.001; Education 2&3: β = 0.595, *p* = 0.076). In the region-stratified analysis, a significant association was observed in the T3 group for participants from rural villages (β = 0.643, *p* = 0.002). The marital status-stratified analysis showed a significant association in the T3 group for married participants (β = 0.589, *p* < 0.001). The smoking-stratified analysis showed a significant association in the T2 and T3 groups for participants who reported smoking (β = 0.419, *p* = 0.034 and β = 0.495, *p* = 0.021). For the episodic memory scores, there is a statistically significant association consistent with the global cognitive scores for T2 or T3 in the following subgroups: males (T2: β = 0.293, *p* = 0.035; T3: β = 0.438, *p* = 0.004), individuals under the age of 60 (T3: β = 0.312, *p* = 0.025), education level 1 (T2: β = 0.335, *p* = 0.005; T3: β = 0.487, *p* < 0.001), individuals with religious affiliation (T3: β = 0.547, *p* < 0.001), married individuals (T2: β = 0.370, *p* = 0.001; T3: β = 0.514, *p* < 0.001), individuals who consume alcohol (T3: β = 0.389, *p* = 0.006), and individuals who smoke or not. No statistically significant associations were observed for mental status scores in any of the stratified analyses.

**Table 4:** Stratified analysis of the relationship between global cognitive function and VAI.

Global cognitive									
	Total			T2			T3		
	β (95% CI)	*p* value	*p* for interaction	β (95% CI)	*p* value	*p* for interaction	β (95% CI)	*p* value	*p* for interaction
Gender			0.086			0.172			0.128
Male	0.107 (0.045, 0.169)	<0.001		0.424 (0.064, 0.784)	0.021		0.598 (0.202, 0.994)	0.003	
Female	0.024 (−0.044, 0.091)	0.494		−0.056 (−0.591, 0.478)	0.836		0.053 (−0.470, 0.577)	0.842	
Age			0.862			0.312			0.884
<60	0.072 (0.020, 0.125)	0.007		0.178 (−0.181, 0.536)	0.332		0.406 (0.039, 0.772)	0.030	
> = 60	0.055 (−0.017, 0.127)	0.132		0.489 (−004, 0.961)	0.052		0.400 (−0.089, 0.889)	0.109	
Education			0.864			0.684			0.984
1	0.090 (0.043, 0.137)	<0.001		0.416 (0.099, 0.733)	0.010		0.567 (0.245, 0.888)	<0.001	
2&3	0.104 (0.001, 0.207)	0.048		0.280 (−0.351, 0.911)	0.384		0.595 (−0.063, 1.254)	0.076	
Region			0.071			0.895			0.190
Rural village	0.109 (0.048, 0.169)	<0.001		0.339 (−0.037, 0.714)	0.077		0.643 (0.245, 1.041)	0.002	
Urban community	0.025 (−0.039, 0.089)	0.448		0.295 (−0.163, 0.753)	0.206		0.210 (−0.240, 0.661)	0.360	
Marry			0.821			0.048			0.077
Married	0.085 (0.040, 0.131)	<0.001		0.441 (0.137, 0.744)	0.004		0.589 (0.281, 0.898)	<0.001	
Others	0.063 (−0.122, 0.248)	0.502		−0.817 (−1.991, 0.358)	0.173		−0.625(−1.909, 0.658)	0.340	
Drinking			0.806			0.437			0.652
Yes	0.096 (0.037, 0.155)	0.002		0.296(−0.056, 0.647)	0.099		0.524 (0.150, 0.898)	0.006	
No	0.075 (0.004, 0.146)	0.039		0.545 (−0.024, 1.114)	0.061		0.619 (0.057, 1.182)	0.031	
Smoke			0.202			0.292			0.365
Yes	0.093 (0.031, 0.155)	0.003		0.419 (0.031, 0.807)	0.034		0.495 (0.075, 0.915)	0.021	
No	0.047 (−0.017, 0.111)	0.147		0.108 (−0.350, 0.565)	0.645		0.289 (−0.162, 0.739)	0.210	

Stratified analysis using GEE methodology. Model adjusted for the cognitive score in 2011, hypertension, diabetes, heart disease, stroke, dyslipidemia, cancer.

**Table 5:** Stratified analysis of the relationship between episodic memory and VAI.

Episodic memory									
	Total			T2			T3		
	β (95% CI)	*p* value	*p* for interaction	β (95% CI)	*p* value	*p* for interaction	β (95% CI)	*p* value	*p* for interaction
Gender			0.232			0.594			0.470
Male	0.082 (0.034, 0.129)	<0.001		0.293 (0.021, 0.565)	0.035		0.438 (0.142, 0.734)	0.004	
Female	0.043 (−0.007, 0.092)	0.089		0.161 (−0.225, 0.547)	0.414		0.271 (−0.106, 0.648)	0.159	
Age			0.585			0.202			0.355
<60	0.057 (0.018, 0.097)	0.005		0.169 (−0.097, 0.436)	0.213		0.312 (0.040, 0.583)	0.025	
> = 60	0.076 (0.022, 0.130)	0.006		0.464 (0.108, 0.820)	0.011		0.532 (0.170, 0.894)	0.004	
Education			0.505			0.832			0.652
1	0.077 (0.042, 0.113)	<0.001		0.335 (0.098, 0.572)	0.005		0.487 (0.247, 0.727)	<0.001	
2&3	0.107 (0.029, 0.185)	0.007		0.399 (−0.100, 0.897)	0.117		0.623 (0.111, 1.135)	0.017	
Region			0.367			0.761			0.327
Rural village	0.084 (0.039, 0.130)	<0.001		0.331 (0.050, 0.612)	0.021		0.547 (0.253, 0.840)	<0.001	
Urban community	0.050 (0.002, 0.097)	0.041		0.253 (−0.086, 0.592)	0.144		0.284 (−0.050, 0.618)	0.095	
Marry			0.670			0.125			0.105
Married	0.078 (0.043, 0.112)	<0.001		0.370 (0.145, 0.596)	0.001		0.514 (0.286, 0.743)	<0.001	
Others	0.046 (−0.084, 0.176)	0.491		−0.276 (−1.158, 0.607)	0.541		−0.169 (−1.199, 0.660)	0.570	
Drinking			0.666			0.095			0.175
Yes	0.073 (0.029, 0.117)	0.001		0.202 (−0.061, 0.466)	0.133		0.389 (0.111, 0.667)	0.006	
No	0.083 (0.030, 0.135)	0.002		0.611 (0.194, 1.028)	0.004		0.696 (0.285, 1.108)	<0.001	
Smoke			0.254			0.894			0.746
Yes	0.081 (0.033, 0.129)	0.001		0.276 (−0.021, 0.574)	0.069		0.401 (0.086, 0.715)	0.013	
No	0.050 (0.004, 0.096)	0.034		0.249 (−0.083, 0.580)	0.141		0.376 (0.048, 0.704)	0.025	

Stratified analysis using GEE methodology. Model adjusted for the cognitive score in 2011, hypertension, diabetes, heart disease, stroke, dyslipidemia, cancer.

**Table 6:** Stratified analysis of the relationship between episodic memory and VAI.

Mental status									
	Total			T2			T3		
	β (95% CI)	*p* value	*p* for interaction	β (95% CI)	*p* value	*p* for interaction	β (95% CI)	*p* value	*p* for interaction
Gender			0.88			0.657			0.749
Male	0.017 (−0.005, 0.038)	0.130		0.040 (−0.094, 0.176)	0.566		0.082 (−0.063, 0.227)	0.269	
Female	0.014 (−0.013, 0.040)	0.31		−0.025 (−0.233, 0.184)	0.82		0.034 (−0.170, 0.238)	0.75	
Age			0.578			0.865			0.499
<60	0.005 (−0.015, 0.024)	0.640		−0.027 (−0.169, 0.114)	0.700		−0.007 (−0.136, 0.149)	0.930	
> = 60	−0.006 (−0.035, 0.024)	0.718		−0.042 (−0.235, 0.152)	0.671		−0.078 (−0.278, 0.123)	0.446	
Education			0.632			0.081			0.228
1	0.005 (−0.014, 0.023)	0.629		0.015 (−0.113, 0.143)	0.820		−0.015 (−0.115, 0.144)	0.827	
2&3	−0.001 (−0.034, 0.031)	0.930		−0.191 (−0.383, 0.000)	0.050		−0.118 (−0.330, 0.095)	0.277	
Region			0.150			0.873			0.420
Rural village	0.006 (−0.018, 0.029)	0.633		−0.041 (−0.193, 0.110)	0.592		−0.018 (−0.178, 0.142)	0.827	
Urban community	−0.012 (−0.035, 0.011)	0.311		−0.045 (−0.211, 0.120)	0.589		−0.066 (−0.228, 0.096)	0.423	
Marry			0.666			0.280			0.380
Married	0.001 (−0.016, 0.018)	0.894		−0.012 (−0.130, 0.106)	0.84		−0.005 (−0.124, 0.115)	0.94	
Others	0.014 (−0.051, 0.080)	0.668		−0.326 (−0.780, 0.127)	0.158		−0.282 (−0.771, 0.206)	0.258	
Drinking			0.897			0.928			0.783
Yes	0.009 (−0.012, 0.030)	0.396		−0.005 (−0.137, 0.128)	0.946		−0.006 (−0.136, 0.148)	0.931	
No	0.009 (−0.018, 0.036)	0.520		0.011 (−0.209, 0.231)	0.920		0.054 (−0.159, 0.266)	0.620	
Smoke			0.71			0.631			0.967
Yes	0.008 (−0.015, 0.030)	0.505		0.018 (−0.131, 0.167)	0.817		−0.013 (−0.164, 0.158)	0.974	
No	0.006 (−0.018, 0.030)	0.630		−0.036 (−0.213, 0.141)	0.690		0.021 (−0.153, 0.194)	0.820	

Stratified analysis using GEE methodology. Model adjusted for the cognitive score in 2011, hypertension, diabetes, heart disease, stroke, dyslipidemia, cancer.

### 3.5. Sensitivity analysis of the association between VAI tertiles and cognitive scores

In the first sensitivity analysis (Table 7), which involved excluding individuals with a history of brain injury, intellectual retardation, or memory-related diseases, we observed significant associations between total VAI and global cognitive function in four Models (β = 0.080, *p* < 0.001; β = 0.070, *p* < 0.001; β = 0.074, *p* < 0.001; β = 0.069, *p* < 0.001), as well as between the T3 VAI tertile and global cognitive function (β = 0.432, *p* = 0.002; β = 0.368, *p* = 0.091; β = 0.390, *p* = 0.005; β = 0.347, *p* = 0.013). Similar interpretations can be made for the remaining sensitivity analyses (Tables 8–10). Additionally, the *p-value* for the trend across the VAI tertiles is provided. For example, in the first sensitivity analysis for global cognitive function across four models (Table 7), we found a statistically significant trend (*p* < 0.001). Similar interpretations can be made for the other sensitivity analyses. Similarly, we observed a similar relationship between VAI and episodic memory. For instance, in the first sensitivity analysis across four Models, we found a positive correlation between Total VAI and episodic memory scores (β = 0.082, *p* < 0.001; β = 0.074, *p* < 0.001; β = 0.078, *p* < 0.001; β = 0.072, *p* < 0.001), as well as between the T3 VAI tertile and episodic memory scores (β = 0.464, *p* = 0.001; β = 0.410, *p* = 0.004; β = 0.434, *p* = 0.002; β = 0.386, *p* = 0.007). The value of p for the trend across the VAI tertiles was less than 0.05 in all four models. Similar conclusions can be drawn from the other sensitivity analyses (Tables 8–10). However, for mental status scores, there was no significant correlation observed with VAI in any of the models or sensitivity analyses (Tables 7–10).

**Table 7:** Sensitivity analysis of VAI and cognitive function (Method 1).

	VAI								*p* for trend
	Total		T1		T2		T3		
	β (95% CI)	*p* value	β (95% CI)	*p* value	β (95% CI)	*p* value	β (95% CI)	*p* value	
*Global cognitive*									
Model 1	0.080 (0.040, 0.119)	<0.001	0	Ref	0.265 (−0.008, 0.537)	0.058	0.432 (0.157, 0.708)	0.002	0.002
Model 2	0.070 (0.031, 0.110)	<0.001	0	Ref	0.215(−0.054, 0.484)	0.118	0.368 (0.091, 0.646)	0.009	0.009
Model 3	0.074 (0.036, 0.113)	<0.001	0	Ref	0.243 (−0.021, 0.506)	0.071	0.390 (0.119, 0.661)	0.005	0.005
Model 4	0.069 (0.029, 0.108)	<0.001	0	Ref	0.231 (−0.032, 0.495)	0.086	0.347 (0.073, 0.622)	0.013	0.013
*Episodic memory*									
Model 1	0.077 (0.044, 0.109)	<0.001	0	Ref	0.303 (0.081, 0.524)	0.007	0.461 (0.238, 0.683)	<0.001	<0.001
Model 2	0.056 (0.024, 0.087)	<0.001	0	Ref	0.205 (−0.009, 0.420)	0.060	0.318 (0.099, 0.538)	0.005	0.005
Model 3	0.058 (0.026, 0.089)	<0.001	0	Ref	0.225 (0.015, 0.436)	0.036	0.329 (0.114, 0.544)	0.003	0.003
Model 4	0.055 (0.023, 0.086)	<0.001	0	Ref	0.221 (0.010, 0.430)	0.040	0.305 (0.087, 0.523)	0.006	0.006
*Mental status*									
Model 1	0.006 (−0.010, 0.023)	0.440	0	Ref	−0.025 (−0.138, 0.088)	0.660	−0.005 (−0.109, 0.119)	0.930	0.928
Model 2	0.018 (0.002, 0.035)	0.032	0	Ref	0.022 (−0.092, 0.136)	0.705	0.083 (−0.033, 0.200)	0.159	0.158
Model 3	0.0190 (0.003, 0.035)	0.023	0	Ref	0.029 (−0.084, 0.141)	0.619	0.089 (−0.026, 0.203)	0.129	0.128
Model 4	0.015 (−0.001, 0.032)	0.077	0	Ref	0.021 (−0.092, 0.133)	0.715	0.062 (−0.055, 0.179)	0.295	0.295

Model 1: Adjusted for the cognitive score in 2011. Model 2: Further adjusted for gender and age. Model 3: In addition to the adjustments in Model 2, further adjustments were made for education, marital status, smoking, and drinking. Model 4: In addition to the adjustments in Model 3, further adjustments were made for hypertension, diabetes, heart disease, stroke, dyslipidemia, liver disease, kidney disease, and digestive system disease.

**Table 8:** Sensitivity analysis of VAI and cognitive function (Method 2).

	VAI								*P* for trend
	Total		T1		T2		T3		
	β (95% CI)	*p* value	β (95% CI)	*p* value	β (95% CI)	*p* value	β (95% CI)	*p* value	
*Global cognitive*									
Model 1	0.082 (0.041, 0.122)	<0.001	0	Ref	0.269 (−0.009, 0.546)	0.058	0.464 (0.183, 0.745)	0.001	0.001
Model 2	0.074 (0.034, 0.115)	<0.001	0	Ref	0.197 (−0.077, 0.470)	0.159	0.410 (0.128, 0.692)	0.004	0.004
Model 3	0.078 (0.039, 0.118)	<0.001	0	Ref	0.218 (−0.050, 0.485)	0.111	0.434 (0.158, 0.709)	0.002	0.002
Model 4	0.072 (0.032, 0.112)	<0.001	0	Ref	0.200 (−0.068, 0.468)	0.143	0.386 (0.107, 0.664)	0.007	0.007
*Episodic memory*									
Model 1	0.077 (0.044, 0.110)	<0.001	0	Ref	0.310 (0.086, 0.535)	0.007	0.478 (0.251, 0.705)	<0.001	<0.001
Model 2	0.058 (0.026, 0.090)	<0.001	0	Ref	0.187 (−0.030, 0.404)	0.091	0.344 (0.121, 0.568)	0.003	0.003
Model 3	0.061 (0.029, 0.092)	<0.001	0	Ref	0.203 (−0.009, 0.417)	0.060	0.359 (0.140, 0.577)	0.001	0.001
Model 4	0.056 (0.024, 0.089)	<0.001	0	Ref	0.197 (−0.016, 0.409)	0.070	0.329 (0.108, 0.549)	0.004	0.004
*Mental status*									
Model 1	0.007 (−0.010, 0.024)	0.420	0	Ref	−0.026 (−0.141, 0.089)	0.660	0.008 (−0.108, 0.125)	0.890	0.891
Model 2	0.018 (0.001, 0.035)	0.033	0	Ref	0.021 (−0.095, 0.137)	0.721	0.085 (−0.033, 0.204)	0.158	0.158
Model 3	0.019 (0.003, 0.036)	0.023	0	Ref	0.023 (−0.091, 0.138)	0.693	0.092 (−0.025, 0.209)	0.123	0.123
Model 4	0.016 (−0.001, 0.033)	0.062	0	Ref	0.013 (−0.101, 0.128)	0.820	0.069 (−0.050, 0.188)	0.255	0.256

Model 1: Adjusted for the cognitive score in 2011. Model 2: Further adjusted for gender and age. Model 3: In addition to the adjustments in Model 2, further adjustments were made for education, marital status, smoking, and drinking. Model 4: In addition to the adjustments in Model 3, further adjustments were made for hypertension, diabetes, heart disease, stroke, dyslipidemia, liver disease, kidney disease, and digestive system disease.

**Table 9:** Sensitivity analysis of VAI and cognitive function (Method 3).

	VAI								*p* for trend
	Total		T1		T2		T3		
	β (95% CI)	*p* value	β (95% CI)	*p* value	β (95% CI)	*p* value	β (95% CI)	*p* value	
*Global cognitive*									
Model 1	0.084 (0.042, 0.125)	<0.001	0	Ref	0.258 (0.023, 0.579)	0.074	0.489 (−0.025, 0.540)	<0.001	<0.001
Model 2	0.076 (0.036, 0.117)	<0.001	0	Ref	0.209(−0.070, 0.487)	0.142	0.433 (0.145, 0.720)	0.003	0.003
Model 3	0.081 (0.041, 0.121)	<0.001	0	Ref	0.230 (−0.042, 0.502)	0.097	0.457 (0.176, 0.738)	0.001	0.001
Model 4	0.073 (0.033, 0.114)	<0.001	0	Ref	0.214 (−0.058, 0.487)	0.123	0.404 (0.119, 0.689)	0.005	0.005
*Episodic memory*									
Model 1	0.077 (0.043, 0.111)	<0.001	0	Ref	0.030 (0.070, 0.526)	0.011	0.484 (0.253, 0.715)	<0.001	<0.001
Model 2	0.058 (0.025, 0.090)	<0.001	0	Ref	0.200 (−0.020, 0.421)	0.075	0.350 (0.122, 0.578)	0.003	0.003
Model 3	0.061 (0.029, 0.093)	<0.001	0	Ref	0.218 (0.008, 0.436)	0.048	0.364 (0.002, 0.434)	0.001	0.001
Model 4	0.055 (0.029, 0.088)	<0.001	0	Ref	0.210 (−0.006, 0.426)	0.057	0.327 (0.102, 0.553)	0.005	0.004
*Mental status*									
Model 1	0.009 (−0.008, 0.025)	0.320	0	Ref	−0.026 (−0.143, 0.092)	0.670	0.027 (−0.092, 0.145)	0.660	0.661
Model 2	0.020 (0.003, 0.037)	0.022	0	Ref	0.021 (−0.098, 0.139)	0.729	0.103 (−0.017, 0.224)	0.093	0.093
Model 3	0.021 (0.004, 0.038)	0.014	0	Ref	0.023 (−0.094, 0.140)	0.701	0.110 (−0.090, 0.229)	0.069	0.069
Model 4	0.018 (0.000, 0.035)	0.042	0	Ref	0.014 (−0.103, 0.131)	0.817	0.086 (−0.035, 0.207)	0.164	0.165

Model 1: Adjusted for the cognitive score in 2011. Model 2: Further adjusted for gender and age. Model 3: In addition to the adjustments in Model 2, further adjustments were made for education, marital status, smoking, and drinking. Model 4: In addition to the adjustments in Model 3, further adjustments were made for hypertension, diabetes, heart disease, stroke, dyslipidemia, liver disease, kidney disease, and digestive system disease.

**Table 10:** Sensitivity analysis of VAI and cognitive function (Method 4).

	VAI								*p* for trend
	Total		T1		T2		T3		
	β (95% CI)	*p* value	β (95% CI)	*p* value	β (95% CI)	*p* value	β (95% CI)	*p* value	
*Global cognitive*									
Model 5	0.064 (0.025, 0.104)	0.001	0	Ref	0.214 (−0.048, 0.476)	0.109	0.330 (0.057, 0.603)	0.018	0.022
*Episodic memory*									
Model 5	0.051 (0.019, 0.082)	0.002	0	Ref	0.213 (0.005, 0.421)	0.045	0.031 (0.074, 0.504)	0.009	0.008
*Mental status*									
Model 5	0.014 (−0.002, 0.031)	0.092	0	Ref	0.018 (−0.095, 0.130)	0.759	0.059 (−0.058, 0.175)	0.440	0.325

Model 5: addition of follow-up time as a confounding factor correction variable in Model 4.

## 4. Discussion

We undertook a longitudinal study to investigate the role of visceral fat accumulation on the evolution of cognitive function. Interestingly, our findings highlighted a correlation between higher VAI scores and improved cognitive abilities among Chinese individuals within the middle-aged and elderly brackets, spanning multiple years. This suggests that an increase in visceral fat might, in fact, have a beneficial influence on cognitive function. Moreover, our research underscores that the primary association of VAI with overall cognition appears to stem from its relationship with episodic memory performance, rather than on mental state. Our discovery of a positive relationship between a heightened VAI and superior cognitive performance stands in stark contrast to prior research, which has largely associated visceral adiposity with cognitive deterioration. This unexpected result challenges the prevailing notion that visceral adiposity uniformly exerts detrimental effects on cognitive health. The potential association of visceral adiposity with better cognitive function observed in our study warrants further investigation.

The relationship between obesity and cognitive function has garnered substantial attention in research. Obesity has been considered numerous underlying causes of diseases including cardiovascular disease, diabetes, and certain cancers for a long time (Wolin et al., 2010; Piché et al., 2020). In recent years, there has been growing interest in investigating the potential link between obesity and cognitive impairment. Several studies have reported an association between obesity and an increased risk of cognitive decline and dementia (Dye et al., 2017). Excess adipose tissue, particularly visceral adiposity, has been proposed as a potential risk factor for the development of cognitive impairment (Mina et al., 2023). Obesity is identified as various vascular risk factors, such as hypertension, dyslipidemia, and insulin resistance, which may lead to chronic brain hypoperfusion, oxidative stress, and inflammation, ultimately damaging brain tissues and compromising cognitive function (Jagust, 2013). However, the relationship between obesity and dementia is not straightforward and has presented a paradoxical finding in some studies. The “obesity paradox” suggests that individuals with obesity may have a lower risk of dementia than those with a BMI (Qizilbash et al., 2015; Tang et al., 2021). The underlying mechanisms behind this paradox are not well understood and may be influenced by factors such as age, sex, genetic susceptibility, and the presence of comorbidities. VAI is a composite indicator that reflects the accumulation of visceral adiposity, VAI has been proposed as a useful tool for assessing the distribution and impact of visceral adiposity on health outcomes (Amato et al., 2010). It provides a quantitative measure that captures the adverse effects of excess visceral fat, which is metabolically active and associated with a higher risk of insulin resistance, dyslipidemia, inflammation, and other cardiometabolic abnormalities (Amato et al., 2010, 2011; Al-Daghri et al., 2013; Jiang et al., 2022; Khanna et al., 2023). In our study, we employed the VAI as a measure of visceral adiposity to investigate its association with cognitive function. Surprisingly, our findings revealed a paradoxical phenomenon, whereby higher VAI scores were positively correlated with cognitive function scores. This unexpected relationship challenges the conventional understanding of the detrimental impact of visceral adiposity on cognitive function.

One possible mechanism is the role of adipose tissue-derived hormones and cytokines, known as adipokines, which have been implicated in regulating inflammation, insulin resistance, and energy metabolism. Adipokines such as adiponectin and leptin may exert neuroprotective effects and promote neuronal growth and survival, potentially contributing to better cognitive function (Fasshauer and Blüher, 2015; Rizzo et al., 2020; Flores-Cordero et al., 2022). Furthermore, the presence of comorbidities often associated with obesity, such as hypertension, diabetes, and dyslipidemia, raises questions about the interplay between these conditions and cognitive health. Individuals with obesity may receive more medical attention and intervention, leading to better management of these comorbidities, which in turn may contribute to preserved cognitive function.

Notably, we found that VAI is positively associated with cognitive function in males, who are under 60 years old. The possible mechanism may reflect processes related to the regulation of androgen. The study has shown that lipolysis was decreased in the androgen-deficient state, in other words, androgen promotes lipolysis (Pecquery et al., 1988; Amengual-Cladera et al., 2012). When men are older than 60 years of age, androgens decrease causing less adipocyte factor. Therefore, a higher VAI is associated with better cognitive function only in men younger than 60 years of age. On the other hand, females are more vulnerable in cognitive function in the old, these may also reflect processes related to the regulation of female hormones. Studies have shown that endogenous estradiol is associated with a decreased risk of cognitive impairment (Yaffe et al., 2000; Bagger et al., 2004). In young women, they have enough endogenous estradiol to protect themselves, while in postmenopausal women, the major source of endogenous estrogen is adipose tissue (Newton et al., 1986; Szymczak et al., 1998). Postmenopausal women need more adipose tissue with higher VAI to decrease the risk of cognitive impairment. Also, factors like metabolism, vascular health, and inflammation, which can be influenced by visceral adiposity, might also play a role in cognitive performance variations with age.

It is important to acknowledge that the obesity paradox is still a topic of ongoing research and debate, and the proposed mechanisms are not yet fully validated. Future studies, employing advanced techniques and longitudinal designs, are needed to unravel the complex interplay between obesity and cognitive health and to identify the underlying molecular, physiological, and behavioral mechanisms.

This study has several strengths worth highlighting. First, we utilized longitudinal data from CHARLS, which provides a comprehensive understanding of cognitive function in the middle-aged and older Chinese population with its extensive representativeness. Second, we employed rigorous statistical analysis methods, including generalized estimating equations and sensitivity analyses, to ensure the reliability and robustness of our results. Additionally, we conducted detailed measurements and categorizations of VAI and cognitive function, allowing for in-depth exploration of the impact of different VAI levels on cognitive function. However, there are limitations to consider in this study. First, our study sample was limited to the middle-aged and older Chinese population, and thus, the generalizability of the findings may be restricted by geographical and cultural factors. Second, our study design was observational, and causality cannot be established. While we conducted multiple adjustments and sensitivity analyses to minimize the influence of confounding factors, there may still be unmeasured potential variables, including factors like mental illnesses, that could impact the results. Moreover, our study primarily relied on self-reported or physician-diagnosed chronic disease information, which may introduce measurement bias.

Further research is needed to overcome these limitations and deepen our understanding of the relationship between VAI and cognitive function. Future studies could incorporate larger sample sizes and multi-center designs, along with objective measures such as biomarkers and neuroimaging, to investigate the biological mechanisms underlying the association.

## 5. Conclusion

In conclusion, our longitudinal study reveals a surprising association between increased VAI and better cognitive function among middle-aged and older Chinese adults. Individuals with higher VAI scores demonstrated higher overall cognitive scores and improved episodic memory performance. These findings challenge the conventional belief that increased visceral adiposity negatively impacts cognitive health. Further research is needed to understand the underlying mechanisms and confirm these unexpected results. These findings have important implications for our understanding of the relationship between visceral adiposity and cognitive function in the Chinese population.

## Data availability statement

Publicly available datasets were analyzed in this study. This data can be found at: http://charls.pku.edu.cn.

## Ethics statement

The studies involving humans were approved by Biomedical Ethics Review Committee of Peking University (IRB00001052-11015). The studies were conducted in accordance with the local legislation and institutional requirements. Written informed consent for participation in this study was provided by the participants’ legal guardians/next of kin.

## Author contributions

ZZ: Formal analysis, Writing – original draft, Conceptualization. KH: Writing – original draft. YC: Formal analysis, Writing – original draft. WJ: Writing – review & editing. YS: Writing – review & editing. LX: Writing – review & editing. FM: Writing – review & editing. GH: Writing – review & editing. YL: Writing – review & editing. XL: Funding acquisition, Supervision, Writing – review & editing.

## Abbreviations

VAI: Visceral Adiposity Index
CHARLS: China Health and Retirement Longitudinal Study
GEE: Generalized Estimating Equation
BMI: Body mass index
TICS: Telephone Interview of Cognitive Status
SD: Standard deviations
CI: Confidence interval
TG: Triglycerides
WC: Waist circumference
HDLC: High-density lipoprotein cholesterol
DSM-5: Diagnostic and Statistical Manual of Mental Disorders
ANOVA: One-way analysis of variance
IQR: Interquartile range
